# HTLV-1 Transmission and HIV Pre-exposure Prophylaxis: A Scoping Review

**DOI:** 10.3389/fmed.2022.881547

**Published:** 2022-04-29

**Authors:** Daniel Bradshaw, Graham Philip Taylor

**Affiliations:** ^1^Virus Reference Department, UK Health Security Agency, London, United Kingdom; ^2^National Centre for Human Retrovirology, Imperial College Healthcare NHS Trust, London, United Kingdom; ^3^Imperial College London, London, United Kingdom

**Keywords:** HTLV-1, PrEP (pre-exposure prophylaxis), antiretroviral (ARV), transmission prevention, integrase inhibitors, nucleoside reverse transcriptase inhibitor (NRTI), PEP (post-exposure prophylaxis)

## Abstract

HIV pre-exposure prophylaxis (HIV-PrEP) is effective in reducing the likelihood of HIV acquisition in HIV-negative people at high risk of exposure. Guidelines recommend testing for sexually transmitted infections (STIs) before starting, and periodically on PrEP, including bacterial infections, HIV, hepatitis C virus, and, for those who are non-immune, hepatitis B virus. Diagnosed infections can be promptly treated to reduce onward transmission. HTLV-1 is not mentioned; however, it is predominantly sexually transmitted, causes adult T-cell leukaemia/lymphoma (ATL) or myelopathy in 10% of those infected, and is associated with an increased risk of death in those without any classically HTLV-associated condition. The 2021 WHO Technical Report on HTLV-1 called for the strengthening of global public health measures against its spread. In this scoping review, we, therefore, (1) discuss the epidemiological context of HIV-PrEP and HTLV-1 transmission; (2) present current knowledge of antiretrovirals in relation to HTLV-1 transmission prevention, including nucleos(t)ide reverse transcriptase inhibitors (NRTIs) and integrase strand transfer inhibitors (INSTIs); and (3) identify knowledge gaps where data are urgently required to inform global public health measures to protect HIV-PrEP users from HTLV-1 acquisition. We suggest that systematic seroprevalence studies among PrEP-using groups, including men who have sex with men (MSM), people who inject drugs (PWIDs), and female sex workers (FSWs), are needed. Further data are required to evaluate antiretroviral efficacy in preventing HTLV-1 transmission from *in vitro* studies, animal models, and clinical cohorts. PrEP delivery programmes should consider prioritizing the long-acting injectable INSTI, cabotegravir, in HTLV-1 endemic settings.

## Background

HIV pre-exposure prophylaxis (PrEP) reduces the likelihood of HIV acquisition and is recommended for high-risk groups. Indications include being HIV-negative and reporting anal or vaginal sex plus any of (1) HIV-positive partners with unknown or detectable viral load, (2) a bacterial sexually transmitted infection (STI), and (3) inconsistent condom use; or injecting drugs, and sharing injecting equipment or injecting with HIV-positive partners (1).

Guidelines recommend STI testing before starting and, periodically on PrEP (1) given the increased risk for STI acquisition. This includes testing for bacterial infections, HIV, hepatitis C virus (HCV), and for non-immune patients, hepatitis B virus (HBV). Diagnosed infections are promptly treated to reduce onward transmission. HTLV testing is not mentioned (1, 2), although HTLV-1 is predominantly sexually transmitted and causes adult T-cell leukaemia/lymphoma (ATL) or HTLV-1 associated myelopathy (HAM) in ~10% of those infected, and is associated with an increased risk of death (Relative Risk (RR) 1.57, 95%CI 1.37–1.80) in those without any classically HTLV-associated condition (3). HTLV-1 is endemic in South America, the Caribbean, Sub-Saharan Africa, Romania, Iran, Japan, and Melanesia. Populations with high seroprevalence are also described within many non-endemic countries. However, data for >2/3 of the global population are lacking (4, 5).

In 2021, the WHO HTLV-1 Technical Report called for the strengthening of public health measures against its spread (6). In this review, we (1) discuss the epidemiological context of HIV-PrEP and HTLV-1 transmission; (2) present current knowledge of antiretrovirals in relation to HTLV-1 transmission prevention; and (3) identify knowledge gaps where data are required to inform the global public health measures to protect HIV-PrEP users from HTLV-1.

## HIV Pre-Exposure Prophylaxis

### Efficacy

The current US Food and Drug Administration-approved HIV-PrEP antiretrovirals are an oral combination of nucleos(t)ide reverse transcriptase inhibitors (NRTI), emtricitabine (FTC) plus either tenofovir disoproxil fumarate (TDF) or tenofovir alafenamide (TAF), taken daily or episodically around a potential exposure, or intramuscular, twice-monthly injections of the long-acting integrase strand transfer inhibitor (INSTI), cabotegravir. Randomized trials have shown the efficacy of TDF/FTC (7, 8) and TAF/FTC (9) PrEP in preventing sexual transmission of HIV-1 (86–97%), with most PrEP failures arising from reduced adherence to oral regimens. The efficacy of injectable cabotegravir is superior (10, 11).

### Characteristics of Groups Using, or Eligible For, HIV-PrEP

Men who have sex with men (MSM) are the major PrEP-user group in industrialized countries. In the UK, 96% of 24,255 users were cis-gender MSM (12). Approximately 1.14 million U.S. adults have indications for PrEP, namely, 71% MSM, 23% heterosexual, and 6% people who inject drugs (PWIDs); notably, 69% were black or Latino (13). In non-industrialized settings, PrEP-user demographics differ. In Kenya, Lesotho, and Tanzania, 77% of 47,352 PrEP-users were women; 50% overall were female sex workers (FSWs) (14).

The MSM and others at risk of HIV acquisition in HTLV endemic countries are increasingly interested in, or already using, PrEP, including Brazil (15), Peru (16), Jamaica (17), and Nigeria (18). In a Brazilian PrEP delivery study, 450/738 (61%) of MSM and transgender women accepted an offer of PrEP (15). Across Brazil, the country with the greatest number of HTLV cases at almost 1 million (19), PrEP demand amongst eligible MSM was estimated at 66,000–98,000 (20). In Japan, modeling suggests that PrEP roll-out would reduce HIV transmission and will likely expand, although prescriptions are not yet widely accessible (21). HIV incidence in indigenous Australians is increasing, a group with one of the highest HTLV seroprevalences in the world (37%, 213/578) (22), and targeted PrEP programmes are expanding (23).

### HTLV-1 Seroprevalence Amongst Groups Eligible for HIV-PrEP

Amongst STI clinic attendees in Jamaica, multiple partners, condomless sex, and a history of STIs or anogenital ulcers were associated with increased HTLV risk (24). HSV-2 seropositivity was associated with increased HTLV-1 seroprevalence amongst UK STI clinic attendees (25).

The HTLV-1 seroprevalence may be increased in MSM in endemic and non-endemic settings (Table 1). In Barbados, 0.7% (1/134) of a predominantly MSM cohort tested HTLV-1 positive at PrEP baseline (26). HTLV risk factors in MSM include HIV (27), multiple partners, condomless receptive anal intercourse (CRAI), syphilis, and HSV-2 (28), a profile that overlaps with that of PrEP-users (29, 30). HTLV-1 seroprevalence in HIV-positive individuals, including MSM, has been reviewed previously and is generally increased compared with that of the general population, indicating either greater exposure or susceptibility (31).

**Table 1:** HTLV-1 seroprevalence studies amongst men who have sex with men.

**Country name**	HTLV-1 seroprevalence in MSM [^*^indicates HTLV untyped]	**Notes**	**HTLV-1 country-specific seroprevalence in the general population, pregnant women or blood donors [ECDC 2015 (4)]**	**Reference**
**HTLV-1 endemic**				
Brazil (Central—Mato Grosso do Sul)	0.7% (3/430)		0.4% (GP)^**^	(32)
Brazil (North—Para)	0.9% 1/107	HIV coinfected	0.4% (GP)^**^	(33)
Brazil (North—Para)	≥1.6% (2/124)	2/2 HIV-1 coinfected. 4 cases of HTLV-2 in MSM also seen. Denominator comprised HIV-infected MSM and heterosexuals.	0.4% (GP)^**^	(34)
Brazil (North East—Ceara)	0.6% (1/171)	HIV-1 coinfected. CSW and PWID.	0.4% (GP)^**^	(35)
Brazil (South East—Campinas, Sao Paulo)	1.4% (8/558)^*^	8/8 HIV uninfected	0.4% (GP)^**^	(36)
Brazil (South East—Sao Paulo)	0.9% (2/229)	2/2 cases HIV-1 coinfected. 1 additional HTLV-2 case (0.4%)	0.4% (GP)^**^	(37)
Brazil (South East—Rio de Janeiro)	4.7% (6/128)	2/6 cases HIV-1 coinfected	0.4% (GP)^**^	(27)
Brazil (South—Rio Grande Do Sul)	2.9% (15/525)^*^		0.4% (GP)^**^	(38)
Brazil (South—Rio Grande Do Sul)	7.0% (5/71)^*^	5/5 HIV-coinfected	0.4% (GP)^**^	(39)
Burkina Faso	4.0 (13/329)^*^		1.0% (PW)	(40)
Dominican Republic	1.5% (1/68)	HIV-uninfected	1.2% (BD)	(41)
Dominican Republic	26.7% (8/30)^*^	All cases were casual sex workers and/or PWID	1.2% (BD)	(42)
Jamaica	4.8% (6/125)	1/6 cases HIV-1 coinfected	2.5% (BD)	(43)
Peru	2.0% (53/2655)	Additionally, 1.3% HTLV-2 (33/2655). Includes 5 (0.2%) with HTLV-1 + HTLV-2 coinfection. 24/86 (27.9%) HTLV cases were HIV-1-coinfected	1.7% (PW)	(44)
Peru	6.2% (3/48)	1/3 cases HIV-1 coinfected	1.7% (PW)	(45)
Peru	2.7% (2/74)*^**^		1.7% (PW)	(46)
Trinidad	15.7% (14/89)	5/14 cases HIV-1 coinfected	1.5% (BD)	(47)
**HTLV-1 non-endemic**				
Argentina	0.4% (3/682)	1/3 HIV-coinfected	0.02-−0.9% (BD)	(48)
Argentina	0.5% (1/200)	HIV uninfected	0.02-−0.9% (BD)	(49)
Argentina	1.3% (1/75)	HIV-1-coinfected	0.02-−0.9% (BD)	(50)
Australia	0.5% (1/200)	1 additional patient was HTLV-1 seropositive by ELISA with insufficient for confirmatory western blot	0% (BD), 33.3% (IAP)	(51)
France	0/32		0.005% (FTBD)	(52)
Indonesia	0.7% (1/143)^*^	HIV uninfected	0% (GP)	(53)
Italy	1.4% (4/285)		0.02% (PW)	(54)
Italy	7.8% (5/64)	5/5 HIV uninfected	0.02% (PW)	(55)
Italy	11.5% (6/52)	6/6 HIV-1-coinfected. All South American male to female transexuals.	0.02% (PW)	(56)
Italy	1.5% (1/66)^*^	HIV uninfected	0.02% (PW)	(57)
Mexico—Tijuana (North West)	1.0% (1/105)^*^		0% (PW), 0.3% (HW)	(58)
Mexico—Guadalajara (West Central)	2.1% 3/146^***^	HTLV ELISA and particle agglutination positive, immunoblot indeterminate. 3/3 HIV-coinfected.	0% (PW), 0.3% (HW)	(59)
Mexico—North East	0/87		0% (PW), 0.3% (HW)	(60)
Mexico—Merida Yucatan (South East)	0/47		0% (PW), 0.3% (HW)	(61)
Mexico—Yucatan (South East)	0/114	12.3% (14/114) were HTLV-2 infected. All HIV-1-coinfected	0% (PW), 0.3% (HW)	(62)
Netherlands	0.4% (3/697)	Further 2/694 developed incident HTLV-1 during follow up. 2/5 were HIV-1 coinfected	0.004% (FTBD)	(63)
Paraguay	3.4% (4/117)^*^		No reliable data	(64)
Singapore	1.6% (1/63)	HIV-uninfected. All CSWs	0.03% (incidence in BD and PW)	(65)
Spain	0/62		0.01% (PW)	(66)
Tahiti	0.6% (1/167)		0% (GP), 0.25% (BD)	(67)
USA—San Francisco	0/349	MSM-PWID cohort	0.005% (FTBD)	(68)
USA—Los Angeles	0/634	HIV infected cohort	0.005% (FTBD)	(69)
USA—Los Angeles	0.08-0.2% (1-3/1290)	1 case HTLV-1; 2 cases untyped. 1 case HTLV-2. All HIV coinfected	0.005% (FTBD)	(70)
USA—Los Angeles	0.08% (1/1276)	HIV-1-coinfected	0.005% (FTBD)	(71)
USA—Washington DC, New York, Hawaii	0/316		0.005% (FTBD)	(71)
USA—Washington DC	1.6% (3/187)	3/3 cases HIV-1 coinfected	0.005% (FTBD)	(72)

* *HTLV untyped*.

#x0002A;*: *800,000/212,600,000 (Brazilian Ministry of Health)(19)*.

*** *HTLV sero-indeterminate*.

*BD, blood donors; FTBD, first time blood donors; CSW, commercial sex worker; GP, general population; HW, healthy women; IAP, indigenous adult population; PW pregnant women; PWID, people who inject drugs*.

Increased HTLV-1 prevalence is reported amongst FSW in endemic settings. A systematic review in Latin America demonstrated greater prevalence than in blood donors or pregnant women, up to 21.8, 0.9, and 1.7%, respectively, in Peru (4, 73). A systematic review in Africa, mostly of endemic countries, identified increased HTLV-1 seroprevalence in FSWs although the difference could have been due to chance (74). In non-endemic settings, HTLV seroprevalence in FSW varies, for example, 0.3% in Spain (HTLV-1) (75) and 6.7% (HTLV-1/2) in the USA (76).

A high HTLV seroprevalence has been reported in PWIDs. HTLV-1 and HTLV-2 seroprevalence amongst HIV-1 positive PWID in Sao Paulo was 15.3 and 11.1%, respectively (37). HTLV-2 seroprevalence amongst U.S. STI clinic attendees was 7.6% vs. 0.7% for PWIDs vs. non-PWIDs, respectively (77). In other non-endemic countries, no HTLV cases among PWID were found, as in Germany (78).

### HTLV-1 as an STI

The rising HTLV seroprevalence observed with increasing age in Japanese and Brazilian cohorts suggests that the major mode of transmission is sexual, with >80% of infections acquired in adulthood (79, 80). Seminal fluid contains T-lymphocytes and macrophages, cells permissive to HTLV infection, and enhances HTLV-1 replication by transactivation of the long terminal repeat promoter (81). Proviral DNA was detected in the cervical fluid of 68% HTLV-1 infected women (82). Phylogenetics has demonstrated sexual transmission within serodiscordant couples (83) and sexual acquisition has been described in other case reports, including subsequent rapidly progressive HAM (84, 85).

Male-to-female transmission is more efficient than vice versa, with a four times greater relative transmission rate (86). Sexual transmission risk per annum is ~0.6–4.9% (86–89). Data are limited on risk for male-to-male contact, for insertive and receptive anal sex. Female-to-female transmission has not been reported.

Reasons for increased efficiency of male-to-female transmission may include enhancing co-factors or greater lymphocyte content within a seminal fluid, inhibitory factors in cervical fluid, or the greater area of susceptible mucosa in the female genital tract. Most seroprevalence studies found that HTLV-1 seroprevalence increases after menopause, suggesting the mucosa becomes more susceptible after this event. Amongst MSM, the association of CRAI with transmission could be explained by the enhancing presence of seminal fluid, or a greater area of susceptible anorectal vs. penile mucosa.

## The Anti-HTLV Activity of Antiretrovirals

### Nucleos(t)ide Reverse Transcriptase Inhibitors

#### Zidovudine

##### In vitro

Zidovudine (AZT) reduced HTLV-1 proviral DNA production following co-culture of infected cell lines with lymphocytes when added at time zero of infection (90–94) but with no impact when added to cells with established infection (91). IC50 was reported as 0.11 μM (94). Enzymatic susceptibility of HTLV-1 and HIV-1 reverse transcriptase (RT) to AZT was equivalent (95).

##### In vivo

In an ATL model, where rabbits were inoculated intraperitoneally with an HTLV-1 transformed cell line, AZT at time zero prevented infection (96). In knockout mice inoculated with human PBMCs and a chronically infected cell line (MT-2), AZT at time zero blocked HTLV-1 infection, but not if given 1 week later (97). In baboons naturally infected with simian T-lymphotropic virus type 1 (STLV-1), treatment with the histone deacetylase inhibitor, valproate, which induces viral expression, followed by AZT, persistently reduced the proviral load (PVL), with a rebound on treatment discontinuation (98). PVL decline was associated with an increase in STLV-1-specific cytotoxic T-lymphocytes. However, AZT did not impact PVL in patients with HAM (99).

#### Zidovudine/Interferon-Alpha (IFN-Alpha)

The combination AZT/interferon (IFN)-alpha improves survival and is the first-line treatment for certain ATL types (100, 101) although the mechanism is uncertain. Although treatment is usually administered indefinitely, cases are described by ATL remission following discontinuation (102). One report demonstrated AZT/IFN-induced inhibition of HTLV-1 RT in responding but not resistant patients with ATL (103), consistent with direct antiviral effects. As RT-mediated viral replication does not occur in malignant cells, this suggests antiviral activity in the ATL microenvironment, either within infected cells or in preventing *de novo* T-cell infection, as such cells are critical for the survival of the malignant clone (104).

#### Lamivudine

##### In vitro

Lamivudine (3TC) protected lymphocytes from HTLV-1 infection *in vitro*, although with reduced potency vs. AZT (105), in one study, 200 times lower (94). However, a methionine-to-valine substitution in the conserved motif of HTLV-1 RT, tyrosine (Y)-methionine (M)-aspartic acid (D)-aspartic acid (D) (YMDD), conferred high-level lamivudine resistance. This motif is homologous with that of HIV-1 RT, where the M184V substitution confers lamivudine resistance, indicating HTLV-1 RT as the target of inhibition (94).

Others reported that lamivudine did not block infection (106) and high-level resistance was observed in an RT enzymatic assay (95). By homology with the reduced susceptibility to 3TC conferred by V118I in association with other HIV-1 RT mutations, HTLV-1 RT resistance to 3TC may be due to the naturally occurring presence of isoleucine at this codon (107).

##### In vivo

Lamivudine therapy in five people with HAM initially reduced the HTLV-1 proviral DNA load by 10-fold, with the nadir reached over a variable period of 1–6 months. However, the effect was not sustained (99).

#### Lamivudine/Zidovudine

AZT-3TC therapy vs. placebo over 48 weeks in 16 patients with HAM had no effect on PVL or clinical response (108) with lack of effect not due to the development of phenotypic NRTI resistance. AZT consistently inhibited HTLV-1 from primary isolates; however, sensitivity was reduced compared to HTLV-1 from MT-2 cells and varied amongst patients. HTLV-1 RT from both primary isolates and MT-2 cells was 3TC-insensitive (109).

#### Tenofovir and Adefovir (Acyclic Nucleoside Phosphonates)

##### In vitro

Inhibition of HTLV-1 cell-to-cell transmission by tenofovir was twenty times more potent in one study than AZT (94). Others reported a similar activity of tenofovir and AZT (110). In cell-free assays, tenofovir inhibited HTLV-1 RT enzymatic activity (93). Others reported the activity of the prodrugs, TDF and TAF, but not of tenofovir, in blocking HTLV-1 cell-to-cell transmission (106). Adefovir and its prodrug, adefovir dipivoxil, approved for anti-HBV therapy, were more active against HTLV-1 than AZT (106). Other groups also observed the blocking of HTLV-1 infection of cells by TDF (111).

##### In vivo

Limited data are available on *in vivo* activity of tenofovir. In a knock-out mouse model, TDF at time zero of inoculation prevented infection, with no effect if administered 1 week later (97). TDF administered for a mean of 9 months in six patients with HTLV-1 had no benefit on PVL or clinical status (109). In the analyses of primary isolates of HTLV-1 RT from patients' peripheral blood mononuclear cells (PBMCs), variable sensitivity to TDF was noted prior to and not altered by AZT/3TC exposure *in vivo*, in contrast with consistent sensitivity to AZT (109).

#### Other NRTIs

Abacavir blocked HTLV-1 infection of cells *in vitro* although less potently than tenofovir (94). The historic NRTIs, didanosine, zalcitabine, and stavudine, were partially active (94, 95, 112). An experimental NRTI class, phosphonated carbocyclic 2′-oxa-3′aza nucleosides (PCOANs), prevented HTLV-1 infection of PBMCs *in vitro* more potently than tenofovir and inhibited the growth of infected cells (109, 110).

### Integrase Strand Transfer Inhibitors

#### *In vitro* 

INSTI, including raltegravir, inhibit HTLV-1 integrase and blocks PBMC infection (113, 114). Barski et al. described INSTI effects against HTLV-1 integrase enzymatic activity and cell-to-cell infection. The EC50 for blocking infection was 0.3, 6.4, and 9.6 nM for bictegravir, raltegravir, and elvitegravir, respectively, and 17.8 nM for TDF. Antiviral activities were comparable with HIV-1 and HTLV-1 integrase (111). Dolutegravir exerted anti-enzymatic activity but potency in blocking infection was not evaluated (111).

#### *In vivo* 

Raltegravir for 12 months reduced the PVL in patients with HAM (*n* = 2) for the first 6 months followed by a rebound, with no impact on the asymptomatic infection (*n* = 3) (115). Genetic substitutions were not observed post-therapy, consistent with a lack of drug selection pressure. Amongst patients with HAM (*n* = 16) receiving 6 months of raltegravir, PVL in PBMC and/or cerebrospinal fluid (CSF) declined in some individuals but with no impact on the overall cohort. Spontaneous lymphoproliferation, a measure of *ex vivo* T-cell activation, was significantly reduced overall. Clinical measurements remained stable (116).

### Other Antiretroviral Classes

#### Non-nucleoside Reverse Transcriptase Inhibitors

The non-nucleoside reverse transcriptase inhibitor (NNRTI), dapivirine intravaginal ring, has been approved by the European Medicines Agency for use as HIV-PrEP in high prevalence settings (117, 118). However, NNRTI is ineffective against HIV-2, a closer relative of HIV-1 than HTLV-1, and nevirapine exerted no activity on HTLV-1 RT *in vitro* (95, 119). This may reflect the greater sequence diversity in RT at the NNRTI-binding pocket than at the substrate-binding site, and the dependence of NNRTI binding on RT structure (120, 121).

#### Protease Inhibitors

Ritonavir inhibits ATL cell growth *ex vivo*, probably due to the anti-nuclear factor kappa-light-chain-enhancer of activated B-cells (NF-kB) activity rather than HTLV-1 protease inhibition (122). The historic HIV-1 protease inhibitor, indinavir, did not inhibit HTLV-1 (123). *In vitro*, darunavir weakly inhibits HTLV-1 protease; novel darunavir analogs have increased activity (124).

#### Entry Inhibitors

The anti-HTLV-1 activity of other antiretrovirals is unknown, including the C-C Motif Chemokine Receptor 5 (CCR5) co-receptor antagonist, maraviroc; fusion inhibitor, enfuvirtide; attachment inhibitor, fostemsavir; or the post-attachment inhibitor, ibalizumab-uiyk. However, a lack of anti-HIV-2 activity of enfuvirtide and fostemsavir suggests inactivity against HTLV-1. A lack of use of a cluster of differentiation 4 (CD4) receptors or CCR5 co-receptors for HTLV-1 attachment predicts the inactivity of ibalizumab and maraviroc, respectively.

### Antiretrovirals: Summary

Data on HTLV-1 susceptibility to NRTIs are conflicting across *in vitro* studies reflecting (1) different assays, particularly comparing cell-to-cell transmission vs. enzyme inhibition, (2) cell-type-dependent drug uptake and intracellular phosphorylation, (3) RT sequence variation, and (4) intracellular RT activity, which may vary with the cellular environment. *In vivo*, most studies have yielded disappointing results for NRTI in established HTLV-1 infection (106). Amongst INSTI, the second-generation agent, bictegravir, showed greater potency in blocking HTLV-1 transmission *in vitro* than TDF. *In vivo*, the antiviral effects of raltegravir in established infection have been disappointing.

The lack of antiretroviral activity in patients probably reflects the greater contribution of HTLV-1-driven cellular proliferation to PVL than viral replication. Extrapolating from HIV-1, a predominance of HTLV-1 transmission through cell-to-cell contacts over virus entry from plasma could reduce RT inhibitor sensitivity, by producing a greater number of infection events per target cell and, therefore, reducing the likelihood of all transmitted viruses being exposed to drugs (106, 125).

NRTI and/or INSTI may be insufficiently potent to treat established HTLV-1 infection. However, the signal for preventing cell-to-cell transmission, mostly marked for AZT, TDF, and bictegravir, suggests a potential role as HTLV-1 PrEP or post-exposure prophylaxis (PEP).

### Current Knowledge of HTLV-PrEP and HTLV-PEP

It has been speculated that following exposure, HTLV-1 proliferation is first through viral replication and infectious transmission, and then predominantly although not exclusively by the mitotic expansion of infected clones (126). However, early infection is asymptomatic and dynamics are poorly understood. The effect of HIV-PrEP is likely through early inhibition of HIV replication, before the infection is established, and similar pre-exposure or post-exposure antiretrovirals with anti-HTLV activity could plausibly prevent HTLV-1 transmission. However, no studies have evaluated HTLV-PrEP, and information on HTLV-PEP is limited to case reports.

## HTLV-PEP

### Organ Transplantation

In iatrogenically immunosuppressed patients infected with HTLV-1 through organ transplantation, any initial phase of infectious spread lasted no more than a few weeks (127). For three organ recipients of one infected donor, AZT/raltegravir was administered from days 17–26 to 43–80 post-transplantation, following the detection of low-level PVL. Peak proviral doubling time was achieved on days 38–45, followed by a steady state. HTLV-1 antibodies were detected on days 16–39. Findings suggested rapid virus dissemination despite antivirals, with both the early infectious spread and mitotic expansion. No rapidly progressive HAM was observed (127).

Elsewhere, AZT/3TC/raltegravir was initiated 24 h post-renal transplantation from an infected donor for 1 month. At PEP cessation, both PVL and HTLV antibodies were undetected. Two months later, high-level PVL and HTLV-1 antibodies were detected, followed by HAM. Antivirals were re-initiated but HAM rapidly progressed (128). The authors speculated that early PEP might delay but not prevent viral propagation. AZT/3TC/raltegravir initiated within 1 week of renal transplantation from another infected donor, and continued for >18 months, did not prevent HAM development in the recipient at 8 months. A second kidney recipient, who received AZT/3TC/raltegravir in the first 2-months post-transplant, and whose allograft was removed following rejection, developed asymptomatic infection over 3 years of follow-up (129).

Consensus, opinion-based guidelines recommend 6 weeks of AZT/raltegravir PEP initiated within 48 h of transplantation from an HTLV-1 seropositive donor; or as pre-emptive therapy, where the donor's HTLV-1 seropositivity is detected >48 h post-transplant and the recipient's PVL is undetected (130). Some clinicians also recommend raltegravir post-stem cell transplant for ATL as this may prevent infection of the donor's lymphocytes; however, data are required to evaluate this approach.

### Neonatal

Although most mother-to-child HTLV-1 transmission (MTCT) occurs *via* breastfeeding, intrauterine and perinatal infection is also reported. Transmission rates are 7-32% for long term (>6 months) breastfeeding vs. 2.5–5.7% for exclusive formula-feeding (131). There are insufficient data on the use of antivirals to prevent MTCT. In UK patients with ATL, MTCT occurred in one case where no antivirals were administered; no MTCT occurred in two cases where maternal AZT/raltegravir plus neonatal AZT were provided (132).

### Occupational

Although occupational exposure, such as in healthcare workers, is a recognized transmission route for HTLV-1 and HTLV-2 (133, 134), data on PEP efficacy are lacking. Some recommend 6 weeks of AZT/3TC/raltegravir after cellular fluid exposure from an HTLV-1 source, especially with a known high PVL (135).

### Sexual

There are no data on antiviral PEP after sexual exposure to HTLV-1.

## Future Directions

HIV-PrEP roll-out has critical implications for HTLV transmission. First, sexually transmitted HTLV outbreaks could occur, as described for HCV (136). Data are urgently required to ascertain the risk and to inform public health interventions. Seroprevalence studies amongst HIV-PrEP-using groups, including MSM, PWID, and FSW, should be performed. In other populations, HTLV seroprevalence may vary considerably even within one country, probably due to founder effects and high transmission rates. Epidemiological findings should inform guidelines indicating country-specific HTLV testing algorithms within PrEP programmes.

Second, PrEP programmes should evaluate antiretroviral efficacy in preventing HTLV-1 transmission. Unlike HIV, antiviral therapy to treat established HTLV is lacking, placing greater emphasis on transmission prevention. Observational cohort studies should measure HTLV-1 incidence in PrEP-users and matched non-PrEP user controls. Cohorts should include MSM and PWID, as most HTLV incidence data are from heterosexual couple studies.

Third, further data on antiretroviral activity against HTLV-1 transmission are required. This should include cabotegravir *in vitro* and in animal models, including in combination with NRTI. Antiretrovirals under development and clinical trials should also be evaluated, particularly, long-acting agents such as the HIV-1 capsid inhibitor, lenacapavir. Data could also be obtained from HBV MTCT prevention studies of TDF, where individuals are HTLV-1-coinfected and compared to non-TDF-receiving controls; however, such studies might only assess the effect of transplacental TDF to prevent intrapartum transmission. In the interim, PrEP programmes should prioritize injectable cabotegravir for roll-out in HTLV-1 endemic settings, both for its predicted greater efficacy in blocking HTLV-1 transmission, and superior efficacy in preventing HIV-1 transmission *vs*. oral NRTI PrEP.

Finally, country-specific data are required to understand HTLV-1 awareness amongst PrEP users, clinicians, and community groups, including risk factors, clinical consequences, treatments for HTLV-related conditions, and measures to prevent onward transmission. Findings should encourage campaigns to improve HTLV-1 knowledge and clinical care.

## Author Contributions

DB and GT devised the manuscript. DB wrote the first draft and GT revised it. Both authors contributed to the article and approved the submitted version.

## Conflict of Interest

The authors declare that the research was conducted in the absence of any commercial or financial relationships that could be construed as a potential conflict of interest.

## Publisher's Note

All claims expressed in this article are solely those of the authors and do not necessarily represent those of their affiliated organizations, or those of the publisher, the editors and the reviewers. Any product that may be evaluated in this article, or claim that may be made by its manufacturer, is not guaranteed or endorsed by the publisher.

## Abbreviations

3TC: lamivudine
ATL: adult T-cell leukaemia/lymphoma
AZT: zidovudine
CCR5: C-C Motif Chemokine Receptor 5
CD4: cluster of differentiation 4
CRAI: condomless receptive anal intercourse
CSF: cerebrospinal fluid
FSW: female sex worker
FTC: emtricitabine
HAM: HTLV-1 associated myelopathy
IFN: interferon
INSTI: integrase strand transfer inhibitor
MSM: men who have sex with men
MTCT: mother to child transmission
NF-kB: nuclear factor kappa-light-chain-enhancer of activated B cells
NNRTI: non-nucleoside reverse transcriptase inhibitor
NRTI: nucleos(t)ide reverse transcriptase inhibitor
PBMC: peripheral blood mononuclear cell
PCOAN: phosphonated carbocyclic 2′-oxa-3′aza nucleosides
PEP: post-exposure prophylaxis
PrEP: pre-exposure prophylaxis
PVL: proviral load
PWID: people who inject drugs
RT: reverse transcriptase
STI: sexually transmitted infection
STLV-1: simian T-lymphotropic virus type 1
TAF: tenofovir alafenamide
TDF: tenofovir disoproxil fumarate.
